# Epidemiology of mental health problems in COVID-19: a review

**DOI:** 10.12688/f1000research.24457.1

**Published:** 2020-06-23

**Authors:** Md Mahbub Hossain, Samia Tasnim, Abida Sultana, Farah Faizah, Hoimonty Mazumder, Liye Zou, E. Lisako J. McKyer, Helal Uddin Ahmed, Ping Ma

**Affiliations:** 1 Nature Study Society of Bangladesh, Khulna, 09000, Bangladesh; 2 Texas A&M School of Public Health, College Station, TX, 77843, USA; 3 Bangladesh Medical Association, Dhaka, 09000, Bangladesh; 4 Gazi Medical College, Khulna, 09000, Bangladesh; 5 United Nations Population Fund, Cox's Bazar, Bangladesh; 6 Ipas, Dhaka, Bangladesh; 7 Exercise & Mental Health Laboratory, School of Psychology, Shenzhen University, Shenzen, 518060, China; 8 National Institute of Mental Health (NIMH), Dhaka, 1207, Bangladesh

**Keywords:** COVID-19, Coronavirus, Mental Health, Mental Disorders, Epidemiology, Review

## Abstract

The novel coronavirus disease 2019 (COVID-19) has become a pandemic affecting health and wellbeing globally. In addition to the physical health, economic, and social implications, the psychological impacts of this pandemic are increasingly being reported in the scientific literature. This narrative review reflected on scholarly articles on the epidemiology of mental health problems in COVID-19. The current literature suggests that people affected by COVID-19 may have a high burden of mental health problems, including depression, anxiety disorders, stress, panic attack, irrational anger, impulsivity, somatization disorder, sleep disorders, emotional disturbance, posttraumatic stress symptoms, and suicidal behavior. Moreover, several factors associated with mental health problems in COVID-19 are found, which include age, gender, marital status, education, occupation, income, place of living, close contact with people with COVID-19, comorbid physical and mental health problems, exposure to COVID-19 related news and social media, coping styles, stigma, psychosocial support, health communication, confidence in health services, personal protective measures, risk of contracting COVID-19, and perceived likelihood of survival. Furthermore, the epidemiological distribution of mental health problems and associated factors were heterogeneous among the general public, COVID-19 patients, and healthcare providers. The current evidence suggests that a psychiatric epidemic is cooccurring with the COVID-19 pandemic, which necessitates the attention of the global health community. Future epidemiological studies should emphasize on psychopathological variations and temporality of mental health problems in different populations. Nonetheless, multipronged interventions should be developed and adopted to address the existing psychosocial challenges and promote mental health amid the COVID-19 pandemic.

## Introduction

The novel coronavirus disease 2019 (COVID-19) caused by the SARS-CoV-2 virus has become a pandemic with a growing number of cases globally ^1^. With the rapid spread of the COVID-19, global health systems are experiencing critical challenges in preventing infections, identifying and managing COVID-19 cases, and ensuring effective strategies to protect public health ^2, 3^. These challenges, although primarily emerging from an infectious disease with physical health implications, may also affect mental health and wellbeing profoundly ^4, 5^. People around the world are grappled with fear and worry about their personal safety, a lack of effective vaccine or treatment, and adverse socioeconomic consequences like unemployment and lack of access to necessary commodities resulting from quarantine and lockdown measures in different contexts ^6, 7^. These issues may have multiple impacts on mental health across populations, which necessitates the attention of global health researchers and practitioners.

Previous studies suggest that depression, anxiety disorders, substance abuse, increased suicidal tendencies, and PTSD commonly follow major economic crises or natural disasters ^8– 10^. If similar patterns hold for the COVID-19 pandemic, the psychological effects of persistence stress among the general population and exacerbation of several mental health disorders among the vulnerable individuals will further strain the current health care system. It may also prevent resumption to normal life for many people when the physical threat to viral infection eventually subsides. The disruption of a normal life as a result of a government-imposed lockdown or stay home orders has significantly impacted the mental health of the affected individuals ^6, 7^. A recent umbrella review of mental health outcomes of quarantine and similar prevention strategies has found that depression, anxiety disorders, mood disorders, posttraumatic stress symptoms, sleep disorders, panic, stigmatization, low self-esteem, lack of self-control are highly prevalent among individuals impacted with physical isolation ^11^. Another rapid review suggested that stressors like prolonged quarantine, fear of infection, frustration, boredom, inadequate supplies, inadequate information, financial loss, and stigma have resulted in long-lasting posttraumatic stress symptoms, confusion, and anger in the mass population ^12^.

The psychological impact of COVID-19 among individuals who are tested positive is another global health concern ^13^. Although the psychological dimensions of COVID-19 are yet to be understood, previous studies suggest that infectious outbreaks critically affect the mental health of the patients who may experience anxiety symptoms, fear, and a lack of hope regarding the uncertainties in treatment and health outcomes ^11^. Several factors influence mental health in this vulnerable population, which include isolation after being diagnosed with the disease, stigma and discrimination, prolonged hospitalization, and a lack of social support ^7, 11, 14^. In COVID-19, these challenges may become more prevalent alongside psychosocial stressors experienced by people in general. Moreover, patients with limited access to healthcare services or those who have preexisting conditions are likely to have higher psychological stress during this pandemic ^15, 16^. Furthermore, multiple mental health problems may have co-existed among individuals and populations even before the onset of the pandemic ^17, 18^, which may increase their susceptibility to adverse mental health outcomes following a COVID-19 diagnosis. Increasing reports and studies inform that patients with COVID-19 may have depression, anxiety disorders, psychological distress, and suicidal behavior ^14, 19, 20^, which necessitates an in-depth understanding of the mental health epidemiology during this pandemic.

COVID-19 may also affect the mental health and wellbeing among healthcare professionals, especially among those who are working as frontline providers ^21, 22^. As COVID-19 cases are impacting the capacities of health systems globally, many of the healthcare providers are working beyond their regular schedules to meet the increased demand for critical care. This makes those healthcare providers susceptible to anxiety, depression, burnout, and insomnia ^22, 23^. A review suggests that physicians face frequent challenges in providing care to their patients, whereas the healthcare systems often mandate the physicians to keep records of their physician-patient encounters along with several clerical responsibilities ^24^, which is likely to increase during this pandemic. Moreover, working without adequate personal protective equipment and other preventive measures increases the fear of contracting the infection, which is becoming a growing concern as a high prevalence of infection among healthcare providers is reported in China, Italy, and the USA ^25– 27^. Furthermore, a lack of social support, working under stress, guilt about suboptimal care to the patient or leaving hospitals understaffed, or and worrying about their families may result in critical mental health challenges among healthcare providers amid COVID-19 ^23, 28– 30^.

In this narrative literature review, we aimed to describe the epidemiology of mental health problems in COVID-19 from available articles retrieved from Medline and Google Scholar using a non-systematic approach. The epidemiological burden of mental health problems is primarily discussed as the prevalence, proportion, or rate of mental health problems from studies that focused on individuals or populations affected by COVID-19. Secondarily, key factors associated with those problems are summarized to inform the risk and protective factors as well as highlight the distributions of epidemiological burden based on variations in those factors. Lastly, we discussed how the current epidemiological evidence might inform future research, policy development, and practice for improving global mental health amid COVID-19 pandemic and future public health emergencies.

## Literature review on the epidemiology of mental health problems in COVID-19

### Prevalence of mental health problems during COVID-19 in different populations

***General population.*** Several studies have reported the burden of mental health problems among the general population during COVID-19 ^13, 19, 31– 42^. For example, a study by Lei and colleagues used self-rating anxiety scale (SAS) and the self-rating depression scale (SDS) to evaluate mental health status among 1593 respondents aged 18 years and above in Southern China ^35^. In this study, the prevalence of anxiety and depression was 8.3% and 14.6%, respectively. Moreover, the prevalence was much higher (12.9%, 22.4%) among individuals who had someone in their social network who had been quarantined compared to the remaining respondents (6.7%, 11.9%). Another cross-sectional study by Liang and colleagues assessed mental health among 584 youths using the General Health Questionnaire (GHQ-12), Negative coping styles scale, and PTSD Checklist-Civilian Version (PCL-C) ^36^. In this study, nearly 40.4% of participants had psychological problems, whereas 14.4% of participants had posttraumatic stress disorder (PTSD) symptoms. A similar study assessed depression and anxiety among 8079 Chinese students using Patient Health Questionnaire (PHQ-9) and the Generalized Anxiety Disorder (GAD-7) questionnaire, respectively ^13^. This study found that the prevalence of depression, anxiety, and a combination of both was 43.7%, 37.4%, and 31.3%, respectively.

Public health interventions like quarantine and isolation may have impacted mental health during COVID-19, which was assessed in several studies. Tang and colleagues assessed PTSD and depressive symptoms in 2485 home quarantined participants from 6 universities using PTSD Checklist Civilian Version and PHQ-9, and data on sleep duration ^37^. This study found that the prevalence of PTSD and depression was 2.7% and 9.0%, respectively. Other mental health problems included feeling extreme fear and short sleep durations.

Mental health at the population level was evaluated using diverse methods. For example, Li and colleagues analyzed Weibo posts from 17,865 active Weibo users using several machine-learning predictive models ^38^. They calculated word frequency and scores of emotional (anxiety, depression, and indignation, for example)) and cognitive (social risk judgment and life satisfaction) indicators before and after the declaration of COVID-19 outbreak on 20 January 2020. This study found that negative emotions like anxiety, depression, indignation, and sensitivity to social risks increased over time, whereas the scores of positive emotions like Oxford happiness and life satisfaction decreased. Another study using the WeChat-based survey among 1577 community-based adults Wuhan, China, used validated GAD-2 (cutoff ≥3) and PHQ-2 (cutoff ≥3) to assess anxiety and depression, respectively ^39^. The prevalence of probable anxiety and depression was 23.84% (95% Confidence Interval [CI]: 21.8–26.0) and 19.21% (95% CI: 17.3–21.2), respectively.

In addition to these cross-sectional studies, a longitudinal study surveyed a total of 1738 respondents from 190 Chinese cities, 333 respondents participated in both sessions of data collection ^40^. This study assessed mental health in the general population two times: first at the beginning of the outbreak and four weeks later, when the outbreak became more widespread. This study found that there was a statistically significant longitudinal reduction in mean Impact of Event Scale-Revised (IES-R) scores (from 32.98 to 30.76, p < 0.01) after four weeks, which was not clinically significant as both scores were above the cut-off for PTSD symptoms. Moreover, the participants had moderate-to-severe stress, anxiety, and depression at the beginning at 8.1%, 28.8%, and 16.5%, respectively, which did not change significantly after four weeks.

While most studies have assessed the mental health impacts during COVID-19 in Chinese populations, fewer studies reported how this pandemic impacted mental health and wellbeing at the population level in other countries. For example, a study conducted in Turkey administered Hospital Anxiety and Depression Scale (HADS) and the Health Anxiety Inventory (HAI) among 343 participants and found that 23.6% of the participants scored above the depression cut-off point whereas 45.1% scored higher than the cut-off point for anxiety ^41^. Furthermore, a study conducted among 662 Indian adults found that more than 80% of the participants were preoccupied with COVID-19 related thoughts ^42^. Moreover, sleep difficulties, paranoia about having COVID-19 infection and distress were reported by 12.5%, 37.8%, and 36.4% participants, respectively. Furthermore, more than 80% of participants in this study perceived a need for mental health services.

COVID-19 has also influenced suicidal behavior among vulnerable individuals in different contexts. Several case studies based on news media reports are found, where individuals committed suicide in fear of COVID-19 ^31– 33, 43^. These cases from India, Pakistan, and Bangladesh have shown how a perceived susceptibility and fear of getting infected has resulted in suicidal attempts among individuals, highlighting a high mental health burden of this pandemic.

***Patients with COVID-19.*** Empirical studies suggest that patients who were tested positive for COVID-19 had experienced adverse mental health outcomes ^14, 19, 20, 29, 44, 45^. A mixed-method study by Guo and colleagues evaluated and compared the mental status and inflammatory markers among 103 patients tested positive with COVID-19 and recruited 103 matched controls that were COVID-19 negative ^14^. This study revealed that COVID-19 patients had higher levels of depression (p < 0.001), anxiety (p < 0.001), and post-traumatic stress symptoms (p < 0.001) compared to non-COVID controls. Moreover, the levels of C-reactive protein (CRP), which is a peripheral inflammatory indicator, positively correlated with the PHQ-9 total score (R = .37, p = 0.003, Spearman's correlation) of COVID-19 patients with symptoms of depression. Moreover, a web-based cross-sectional survey among 7,236 Chinese individuals identified the overall prevalence of GAD, depressive symptoms, and deteriorated sleep quality as 35.1%, 20.1%, and 18.2%, respectively ^29^. Furthermore, suicidal behavior among individuals who were tested positive for COVID-19 is another mental health concern. A case from India suggests that COVID-19 may critically impact psychosocial wellbeing and influence suicidal attempts among the affected individuals, which may also aggravate if the patient has other comorbid diseases ^19^.

COVID-19 may have impacted psychological health among COVID-19 positive individuals with preexisting mental health problems differently than patients with no such history. This was assessed in a study by Hao and colleagues, who evaluated mental health among 76 psychiatric patients and 109 healthy controls from Chongqing, China using the IES-R for PTSD symptoms, Depression, Anxiety and Stress Scale (DASS-21) for anxiety, depression, distress, and Insomnia Severity Index (ISI) for sleep disorders ^44^. In this study, the mean scores in IES-R, DASS-21 subscales, and ISI were significantly higher among psychiatric patients than healthy controls (p < 0.001). Moreover, worries about their health, anger issues, impulsivity, and suicidal ideation were higher in psychiatric patients compared to the healthy controls (p < 0.05). About one-third of psychiatric patients fulfilled the diagnostic criteria for PTSD, and more than one-quarter of the recruited psychiatric patients had moderately severe to severe insomnia. This study informs that the severity of adverse psychological effects on psychiatric patients during the COVID-19, which similar to another study by Bo and colleagues ^45^.

Multiple primary studies synthesized in reviews provide collective insights on the epidemiological burden. A recent meta-analytic review of 12 studies assessed mental health among 976 patients with COVID-19 ^20^. More of the cases were from Wuhan and other cities in China. These studies provided evidence for neuropsychiatric problems, including delirium. For example, confusion was observed in 65% of the intensive care unit patients, whereas agitation was found in 69% of the intensive care unit patients in one study. Moreover, altered consciousness in 21% of patients who subsequently died as reported in another study. During discharging from the hospital, 33% of COVID-19 patients in a study were experiencing the dysexecutive syndrome.

***Healthcare providers.*** Several studies have evaluated the burden of mental health problems among healthcare providers during COVID-19 ^21, 28, 46– 55^. In a single-center cross-sectional survey using a numeric rating scale (NRS) on fear, Hamilton Anxiety Scale (HAMA), and Hamilton Depression Scale (HAMD), 2299 participants were enrolled, including 2042 healthcare providers and 257 administrative staff from the same institution ^48^. In this study, significant differences in the severity of fear, anxiety, and depression were observed between the two groups. In addition, frontline healthcare providers with close contact with COVID-19 patients were 1.4 times more likely to experience fear and nearly twice more likely to experience anxiety and depression compared to the non-clinical staff. Another study by Cao and colleagues found 6.3% of the participating doctors felt nervous after listening to the news on mass media that some doctors were positive for COVID-19, whereas 52.6% of the participating nurses reported negative emotions, worrying about family, fear of infection, and stress about heavy workload ^49^. Moreover, a study from Wuhan assessed mental health in 105 healthcare providers who were positive for COVID-19, which found that 88.3% of the participants experienced either psychological stress or emotional changes during the isolation period ^50^. Furthermore, a comparative assessment by Chen and colleagues found that the mean SDS and SAS scores were significantly higher among Chinese healthcare providers than that of the general Chinese population (47.1 ± 10.5 vs. 41.9 ± 10.6 for SDS; 40.3 ± 11.5 vs. 29.8 ± 10.1 for SAS; *Ps* < 0.001) ^51^.

Similar studies in different institutions provide a detailed overview of how COVID-19 has impacted mental health among healthcare providers. A study conducted among 120 healthcare provider medical staff used Symptom Checklist 90 (SCL-90), SAS, SDS, and PCL-C to assess their mental health ^52^. Among the study participants, 60 staff from COVID-19 designated hospitals were assigned to the experimental group, and 60 staff from the non-designated hospital were assigned to the control group. This study found that the SCL-90 scores of somatization, depression, anxiety, and terror were much higher among the staff at the COVID-19 designated hospital. Moreover, the SAS (45.89±1.117), SDS (50.13±1.813), and PCL-C (50.13±1.813) scores were significantly higher in the experimental group than the control participants. Furthermore, the average PSQI score in the experimental group was 16.07±3.761, which informed that the sleep quality was poor among those participants, whereas 61.67% and 26.67% have moderate and severe insomnia, respectively. Another study by Wu and colleagues, the frequency of burnout was assessed and compared between 220 physicians and nurses on the frontline wards and those working in the usual wards using the Maslach Burnout Inventory-medical personnel ^53^. The findings of this study suggest that the frontline participants had a lower frequency of burnout (13% vs. 39%; p < 0.0001) and were less worried about being infected with coronavirus compared with participants from usual wards. This suggests that working as a frontline provider may not be an independent risk factor for mental health problems. Rather, healthcare providers from both frontline and usual wards may have unique mental health needs and both groups should be considered for preventive mental health measures amid this pandemic.

Similar studies have shown a high prevalence of multiple mental health problems across contexts. For example, a cross-sectional survey in Gansu, China, assessed mental health among 79 doctors and 86 nurses with a questionnaire set consisted of the SAS, SDS, and the simplified coping style questionnaire (SCSQ) ^54^. The prevalence rates of anxiety and depression symptoms among doctors were 11.4% and 45.6%, respectively, whereas the prevalence among nurses was 27.9% and 43.0%, respectively. Moreover, Li and colleagues conducted a cross-sectional study in 948 medical staff in Wuhan and Ningbo, China ^55^. In this study, the medical staff in Wuhan had higher insomnia than in Ningbo (58.90 vs. 24.97%; p = 0.001) and had higher general psychological symptoms (13.24 vs. 8.64%; p = 0.044). Moreover, Li and colleagues conducted a study among 4369 female healthcare providers. The prevalence of depression, anxiety, and acute stress symptoms among the participants was 14.2%, 25.2%, and 31.6%, respectively ^21^. Furthermore, Tan and colleagues assessed psychological effects on the workforce returning to work during the COVID-19 using the IES-R, DASS-21, and ISI scales ^46^. Among the study participants, 10.8% had PTSD symptoms after returning to work. Furthermore, participants in this study had a prevalence of anxiety disorders (3.8%), depression (3.7%), stress (1.5%), and insomnia (2.3%).

Another study by Kang and colleagues evaluated the mental health status of 994 medical and nursing staff in Wuhan ^28^. In this study, 36.9% of the participants had subthreshold mental health disturbances (mean PHQ-9: 2.4), 34.4% had mild disturbances (mean PHQ-9: 5.4), 22.4% had moderate disturbances (mean PHQ-9: 9.0), and 6.2% had severe disturbance (mean PHQ-9: 15.1) in the immediate wake of the viral epidemic. Studies outside China have shown a similar burden of mental health problems among the healthcare providers during COVID-19. For example, Chew and colleagues assessed mental health among 906 healthcare workers in Singapore and India ^47^. Among the study participants, 48 (5.3%) were screened positive for moderate to very-severe depressive symptoms, 79 (8.7%) had moderate to extremely severe anxiety symptoms, 20 (2.2%) had moderate to extremely severe stress, and 34 (3.8%) had moderate to severe levels of psychological distress. Moreover, a headache was the commonest reported symptom (32.3%), whereas 33.4% of participants reported having more than four neuropsychiatric symptoms.

### Factors associated with mental health problems during COVID-19

***Age.*** Several studies reported younger age as a risk factor for mental health problems amid COVID-19 ^28, 29, 56^. For example, Huang and colleagues found that younger people had a significantly higher prevalence of generalized anxiety and depressive symptoms compared to older people ^29^. Moreover, Wang and colleagues reported the anxiety risk of people above 40 years old was 0.40 times (95% CI: 0.16–0.99) than participants aged below 40 years, suggesting that younger individuals had a higher risk of anxiety ^56^. However, Chew and colleagues found mental health problems as more prevalent among older adults, contradicting studies discussed earlier ^47^.

***Gender.*** Female gender was reported as a common risk factor for mental health problems in several studies ^13, 21, 28, 41, 55– 57^. For example, Li and colleagues reported that female patients were more likely to have anxiety (OR = 3.206, 95% CI: 1.073–9.583, p < 0.05) and depression (OR = 9.111, 95% CI: 2.143–38.729, p < 0.01) compared to male patients ^57^. In another study by Guo and colleagues, female patients with COVID-19 had a higher perceived helplessness compared to male patients (Z = 2.56, p = 0.010), and controls of both female (Z = 2.37, p = 0.018) and male genders (Z = 2.87, p = 0.004) ^14^. Another study among medical staffs in Wuhan conducted by Li and colleagues found that the symptoms of insomnia were significantly related to female gender (OR = 1.379, p = 0.042, 95% CI: 0.65–2.17) ^55^.

***Marital status.*** Marital status was associated with mental health status among individuals experiencing mental health problems during COVID-19. Li and colleagues found that insomnia was related to marital status (OR = 0.57, p = 0.046, 95% CI: 0.33–0.99) among medical staff in Ningbo, China ^55^. Another study by Tan and colleagues reported that the severity of psychiatric symptoms in the workforce returning to the workplace was significantly associated with marital status (p < 0.05) ^46^.

***Education.*** Several studies have reported education as an associated factor of mental health status and problems during the COVID-19 pandemic ^13, 35– 37, 55, 56^. For example, Liang and colleagues found that mental health among young participants was significantly related to being less educated (OR = 8.71, 95% CI: 1.97–38.43) ^36^. Also, Lei and colleagues reported that low education was associated with poor mental health outcomes ^35^. In contrast, Zhou and colleagues found that students in senior high school and those with higher grades had a greater prevalence of depressive and anxiety symptoms ^13^. Similarly, Wang and colleagues reported that those with a bachelor's degree group had a depression risk of 0.39 times (95% CI: 0.17-0.87) compared to participants with a master's degree or above ^56^. This evidence highlights that education may have some protective roles as seen in the study by Lei or Liang and colleagues, but later studies emphasized that the added academic stress may affect mental health amid this pandemic.

***Occupation and income.*** Empirical studies reported associations of occupation, income, and economic conditions with susceptibility to mental health problems ^21, 29, 35, 36, 56^. For example, Huang and colleagues found that healthcare workers were more likely to have poor sleep quality compared to other occupational groups ^29^. Another study by Liang and colleagues found that participants working as an employee in the local enterprises (OR = 2.36, 95% CI: 1.09-5.09) had an elevated risk of poor mental health outcomes compared to other occupational groups ^36^. Also, lost economic opportunities due to lockdown were reported in a suicide case of a farmer in India, which informs socioeconomic challenges that may critically impact mental health among marginalized individuals ^43^. Moreover, people with property loss and adverse economic conditions are more vulnerable to mental health problems ^35^. These highlight the impacts of occupational stress as well as economic instability or challenges in mental health during COVID-19.

***Place of living and close contact with people with COVID-19.*** In several studies, the location or place of living was associated with mental health problems among study participants ^35, 41, 55^. For example, Özdin and colleagues reported that living in urban areas was associated with depression ^41^. Moreover, Lei and colleagues found that individuals living in Chongqing had higher anxiety and depression scores than those living in Yunnan Province ^35^, highlighting two cities in the same country may have different levels of associations with mental health among residents living in those cities. Another study by Li and colleagues found that patients with contact history and people in the epidemic area were more likely to experience depression (OR = 3.267, 95% CI: 1.082-9.597, p <0.05) ^55^. Moreover, close contact with COVID-19 patients impacted mental health and wellbeing. For example, suspected or confirmed cases among family members or relatives were significantly associated with depressive symptoms (OR = 1.93; 95% CI: 1.29-2.86; p = 0.001) among women healthcare providers ^21^, which added psychosocial burden in addition to their occupational stress in hospitals where they spent the most time during COVID-19.

***Comorbid physical health problems.*** The presence of comorbid physical health problems like diabetes, cerebrovascular diseases, heart diseases and other chronic conditions as a risk factor associated with mental health problems amid COVID-19 in several studies ^21, 41, 44, 46, 47^. For example, accompanying chronic diseases were identified as risk factors for anxiety during COVID-19 in a study by Özdin and colleagues ^41^. Another study by Chew and colleagues found that comorbid physical symptoms experienced in the preceding month were significantly associated with depression (OR = 2.79, 95% CI: 1.54-5.07, p = 0.001), anxiety (OR = 2.18, 95% CI: 1.36-3.48, p = 0.001), stress (OR = 3.06, 95% CI: 1.27-7.41, p = 0.13), and PTSD (OR = 2.20, 95% CI: 1.12-4.35, p = 0.023) among the study participants ^47^.

***Comorbid mental health problems.*** People with preexisting mental health problems are highly vulnerable to experience psychological impacts of COVID-19 ^21, 35– 37, 41, 44, 47, 54, 55^. For example, Hao and colleagues found that comorbid psychiatric illness was significantly associated with higher mean IES-R, DASS depression, anxiety, and stress subscale scores and ISI scores (p < 0.05). The findings of this study inform that the severity of negative mental health impacts was higher among individuals with a clinical history of psychiatric comorbidity ^44^. Also, people with substance use disorders are susceptible to COVID-19 ^58^, which may also increase their vulnerability to subsequent mental health problems. In another study, Liang and colleagues found that suffering from PTSD symptoms was significantly associated with mental health (OR = 1.05, 95% CI: 1.03-1.07) among young participants ^36^. Moreover, Zhu and colleagues reported that a history of depression or anxiety was a significant risk factor for doctors (T = -2.644, p = 0.010, 95% CI: -10.514~-1.481), which was also associated with an increased risk of anxiety symptoms (T = -3.635, p = 0.000, 95% CI: -16.360~-4.789) and depression symptoms (T = -2.835, p = 0.005, 95% CI: -18.238~-3.254) among the participating nurses ^54^.

***Exposure to COVID-19 related news and social media.*** Several studies have reported that exposure to COVID-19 related social media contents or mass media news was associated with adverse mental health outcomes amid COVID-19 ^29, 39^. Ni and colleagues found that spending more than 2 hours per day on COVID-19 related news via social media was associated with anxiety and depression among community-based adults ^39^. Another study by Huang and colleagues reported that time spent focusing on the COVID-19 for equal or more than 3 hours per day was associated with generalized anxiety disorder ^29^.

***Coping styles.*** In COVID-19, mental health outcomes among individuals were associated with their coping skills. For example, Liang and colleagues assessed the magnitude and predictors of mental health problems among youth and found that negative coping styles were significantly associated (OR = 1.03, 95% CI: 1.00–1.07) with adverse mental health outcomes ^36^. Similar findings from Zhu and colleagues suggest that the total score of positive coping was negatively correlated with the total score of anxiety (r = -0.182, p = 0.002) and depression (r = -0.253, p = 0.001) among individuals affected by the COVID-19 pandemic ^54^.

***Psychosocial support.*** Psychosocial stressors during COVID-19 had low impacts on individuals who had better psychological and social support from their family and social networks ^35, 39^. Lei and colleagues found that individuals with no psychosocial support were highly vulnerable to anxiety and depression during this pandemic ^35^. Moreover, Ni and colleagues reported that social support was associated with a lower risk of anxiety and depression among both health professionals and community-based adults in their study sample ^39^.

***Other factors associated with mental health impacts during COVID-19.*** Several other factors associated with the mental health impacts of COVID-19 has been reported. For example, Wang and colleagues reported that a high level of confidence in doctors, perceived likelihood of survival, personal protective measures, satisfaction with health communication, and a low risk of contracting COVID-19 minimized the risks of adverse mental health outcomes during COVID-19 ^40^. Similarly, Tan and colleagues found that personal prevention measures including hand hygiene and wearing face masks and organizational measures, including improvement of workplace hygiene and proactive approaches from the company, were associated with a significantly lower risk of mental health problems (p < 0.05) among healthcare providers ^46^. In addition, stigma to COVID-19 was a major challenge associated with adverse mental health outcomes and events, including suicidal behavior ^14, 59^. Moreover, exercise habit was found as a common protective factor of depression, anxiety, and acute stress symptoms in female healthcare providers in a study by Li and colleagues ^21^. Furthermore, participants in this study reported that working in other departments (OR = 0.78; 95% CI: 0.65 to 0.94; p = 0.009) was a protective factor of mental health during COVID-19. Among the study participants, those who worked in the isolation wards had more resigning thoughts (16.9% vs. 7.8% in other departments; p < 0.001) and life-threatening thoughts once infected (67.7% vs. 56.4% in other departments; p < 0.001), which suggests that the placement within workplace had significantly impacted mental health outcomes among the participants.

## Discussion

### Overview of the epidemiology of mental health problems in COVID-19

This review evaluated and narratively synthesized available scholarly articles on the impacts of COVID-19 on mental health in different populations alongside factors associated with mental health conditions in those studies. The available literature suggests that people have a wide range of mental health problems, including depression, anxiety disorders, stress, panic, anger, impulsivity, somatization disorder, sleep disorders, emotional disturbance, PTSD, suicidal behavior, and so on ^14, 21, 28, 35, 37, 41, 44, 46, 47, 60^. Studies have shown that the general population who had been experiencing different levels of psychosocial stressors amid the COVID-19 pandemic had developed mental health problems ^13, 31– 42^. In addition to general stressors, a fear of ongoing outbreak, susceptibility to infection, exposure, or close contact with someone with COVID-19 affected mental health and wellbeing among the general population ^35, 41^. Moreover, individuals with COVID-19 diagnosis had profound psychological distress, anxiety, depression, and other mental health problems compared to those who were not infected ^14, 19, 20, 29, 44^. This illustrates the fear of adverse health outcomes due to COVID-19 may have affected mental health, which also highlights the mental health aspect of a physical health problem among those individuals. Furthermore, healthcare providers working as frontline workers or other departments had varying levels of psychosocial burden amid this pandemic ^28, 46– 55^. For those workers, prolonged exposure or contact to persons with COVID-19, preexisting psychosocial challenges, and low institutional and social support contributed to the deterioration of mental health status ^28, 46, 47, 50, 52^. This review also found several socio-demographic and psychosocial factors associated with mental health problems in the COVID-19 pandemic. People of younger and older age had different risks of developing mental health problems ^28, 56^, whereas women were at a higher risk of such problems compared to men ^13, 41, 56, 57^. Moreover, marital status, education, and economic challenges, including unemployment, loss of income, or economic opportunities due to lockdown or other social measures were associated with mental health outcomes ^36, 41, 46, 60^. Furthermore, the place of residence or living near outbreak areas impacted mental wellbeing ^41, 55^, whereas comorbid physical and mental health problems made individuals highly susceptible to mental health problems during this pandemic ^35– 37, 41, 44, 54, 55^. In addition, spending more time on social media content or news related to COVID-19, lack of social support, stigma, inadequate personal preventive measures, and working in COVID-designated departments were associated with a high risk of mental health problems amid this pandemic ^14, 29, 39, 46, 61^. These findings on mental health burden and associated factors inform several critical insights on the psychosocial epidemiology of this pandemic alongside unique perspectives on how to mitigate mental health problems through future research, policymaking, and practice.

### Implications for future mental health research in COVID-19

***Understanding the psychopathological variations in COVID-19.*** Empirical studies evaluating mental health amid COVID-19 have shown how different factors associated with mental health problems impacted individuals and populations. However, those factors may not affect everyone in a similar way. For example, people with existing mental disorders or poor socioeconomic challenges may have suboptimal mental health status ^15, 17^, which highlights the need for a comprehensive assessment of risk factors for specific mental health problems alongside a holistic assessment of overall mental health and wellbeing in those individuals and populations. Moreover, individuals may have different levels of psychological resilience and coping mechanisms to stressful events, which may result in different mental health outcomes among individuals despite having similar exposure to psychosocial stressors ^62^. These unique variations are evident in the existing literature on COVID-19 and mental health, which necessitates more research on psychopathological processes and outcomes in individuals and populations globally.

***Evaluating the temporality of mental health impacts of COVID-19.*** Most studies available on COVID-19 and mental health are cross-sectional in nature, which may not inform the incremental changes in mental health outcomes among the affected individuals. Although some studies recruited matched controls or compared with the earlier mental health status of the individuals ^14, 52^, there is a critical need to evaluate such changes using longitudinal study designs. Moreover, such studies should evaluate how different risk factors contribute to psycho-pathogenesis in diverse populations in different contexts over time.

***Improving measurements and methodological approaches.*** Studies in the review have used a wide range of methods and instruments for measuring mental health, which included SAS, SDS, GHQ, PHQ, GAD-7, IES-R, HADS, HAMA, and HAMD. Many of these are widely used across mental health studies ^63^; however, the reliability and validity of these instruments in the context of a pandemic affecting almost every aspect of human life remain unknown. In addition, the meanings of sub-scales or questions in each instrument may appear to be different from the individuals who experienced profound psychosocial stress during this pandemic. One consideration for future research can be focused on the development and adoption of instruments that may provide more accurate measurements of mental health and psychosocial constructs in this pandemic as well as other public health emergencies.

***Promoting multi-disciplinary mental health research.*** COVID-19 has brought unique challenges to all aspects of health and wellbeing, which include social, cultural, educational, occupational, economic, political, and other dimensions of human lives ^7^. This phenomenon is evident in the findings of this review as well; a wide range of factors associated with mental health problems suggest that those should be examined using multi-disciplinary approaches enabling researchers from different disciplines to bring their unique insights and resources to better understand the social determinants of mental health in the era of a pandemic. Although health systems remain on the focus in such research efforts, other social systems and stakeholders should be engaged in future mental health research in COVID-19 and similar disruptive global events ^64, 65^.

***Emphasizing on research and evidence mapping on mental health.*** This review found a high burden of different mental health problems reported in empirical studies, case studies, and reviews. However, it cannot be concluded that the current evidence illustrates the true burden of mental health problems for several reasons. One such reason is a critical lack of studies from Africa, the Americas, and South Asia, which informs a lack of mental health research during COVID-19 in those contexts. Perhaps, those countries started having COVID-19 cases after China and European countries, which could be a reason for delayed research and scholarly publications on mental health during COVID-19. Another challenge is the rapidly evolving literature on COVID-19 that provides early stage of research evidence ^66^. As more primary studies become available, it will be necessary to perform a systematic appraisal and synthesis of the evidence of those studies for effective decision-making. These issues should be addressed through strengthening research capacities in resource-constrained contexts and facilitating rigorous evidence synthesis in global mental health.

### Implications for mental health policymaking and practice

***Developing effective mental health interventions and strategies.*** The current evidence on the epidemiological burden of mental health problems in COVID-19 necessitates the development and implementation of multipronged interventions and strategies addressing the same. In this discourse, the mental health needs in vulnerable populations, including those with preexisting physical and mental health problems, should be assessed and managed carefully. Moreover, as in-person mental health services are most disrupted, psychosocial interventions offered through digital platforms like the internet, social media, mobile phones, and apps are increasingly being adopted ^67^. However, the effectiveness, safety, and quality of such digital mental health interventions should be assessed prior to recommending those in practice ^68^. Moreover, the digital divide in different population groups like those who have low education, old age, or living in a rural area with limited access to technologies may not use these services ^69^. Therefore, context-specific strategies allowing multiple ways for access to mental health should be ensured. In this regard, mental health policies and programs should be revisited and strengthened, considering the operational challenges amid COVID-19.

***Prioritizing and integrating mental health in existing systems of care.*** A high prevalence of mental health problems inform a widespread need for mental health services, whereas most countries lack adequate infrastructure and human resources to provide the same. Earlier, scholars and practitioners have argued about integrating mental health services in primary care ^70^, which may substantially improve access to mental health services globally. Another way to improve mental health care can be strengthening community-based and social health programs and incorporating mental health components with the provision of referral care whenever required ^71^. The appropriateness of such approaches should be assessed for each context and adopted, ensuring infection prevention strategies complementing those approaches.

***Improving access to information and mental health resources.*** Many studies highlighted the fact that access to accurate information was associated with a lower risk of mental health problems. Since the beginning of the pandemic, rumors or misinformation had emerged in mass and social media platforms ^72^. This highlights inadequate health communication amid this pandemic, which might have induced fear and affected mental health globally. It is essential to ensure effective health communication regarding the facts and preventive measures in a timely way to prevent public anxiety and fear of COVID-19 ^72^. Moreover, access to resources promoting positive mental health can substantially help in addressing and self-managing mental health challenges among individuals ^73^. Online resources like self-help meditation, mental health education, information on early signs, and caregiving could be helpful, which should be considered for preventing COVID-19 and associated mental health problems.

***Addressing mental health inequities.*** Several social determinants of mental health are identified in the current literature, which emphasizes the roles of the structural inequities in different contexts that influence psychosocial stressors and overall mental health outcomes among the affected individuals. Therefore, addressing inequities in the social determinants of mental health should be a long-term goal of future policymaking and mental health practice ^74^. Such efforts, if adopted, might help in developing resilience to mental health challenges, preventing mental disorders, and promoting positive mental health at the population level.

***Mobilizing social and community resources and organizations.*** Public health challenges necessitate mobilizing resources available within and between contexts. In COVID-19, psychosocial challenges experienced by individuals may be addressed by local and regional organizations through enhancing collaboration and extending social capital. Mobilizing two types of social capital, i.e., bonding capital within a context and bridging capital from another context, may help communities to improve their psychosocial wellbeing and other factors associated with mental health ^75^, which can be considered as a strategy amid the COVID-19 pandemic.

***Strengthening mental health systems for COVID-19 and future public health emergencies.*** Health systems around the world have experienced a critical lack of preparedness in fighting COVID-19, which includes the mental health impacts of this pandemic as well. Most nations do not have strong mental health systems that may ensure a continuum of mental health care from prevention to institutional care for severe mental disorders; therefore, those systems may not address the added burden of mental health problems in this pandemic. Perhaps, one of the greatest lessons from COVID-19 would be strengthening mental health systems ensuring resilience to systematic shocks as seen during public health emergencies ^11^. Potential strategies to achieve such resilience may include establishing mental health policies, developing population-based programs, enhancing institutional capacities to develop mental health workforce, revising health systems financing for mental health, engaging communities, and institutions to address barriers to access to mental health and promote positive mental health across populations.

### Limitations of this review

There are several limitations of this review, which should be acknowledged. First, this is not a systematic review, which makes this review subject to selection bias while searching for databases without a structured format. For the same reason, a rigorous appraisal and synthesis of the global evidence are not done in this review that affects the quality of evidence presented in this study. Second, this review did not focus on a specific mental health problem, which could have provided a more in-depth analysis of the literature on that issue. Third, this review did not focus on context-specific correlates of mental health problems separately, which could have facilitated local-level decision making. Despite these challenges, this narrative review provides a comprehensive overview of mental health epidemiology in COVID-19 based on the early-stage research findings. The limitations of this review should be addressed in future reviews adopting a more systematic approach with the emergence of more epidemiological studies, which would inform substantially better evidence for policymaking and practice.

## Conclusion

COVID-19 is a global public health emergency with enormous impacts on mental health. This narrative review found that individuals affected in the pandemic may have a high epidemiological burden of depression, anxiety disorders, stress, panic attack, somatization disorder, sleep disorders, emotional disturbance, PTSD symptoms, suicidal behavior, and many more mental health problems. Moreover, a wide range of demographic and psychosocial factors are associated with mental health problems during this pandemic that highlights some people who are especially vulnerable to those adverse outcomes. Taken together, the epidemiological distribution of mental health problems informs a psychiatric epidemic is cooccurring with the COVID-19 pandemic, which is evidently becoming a global health challenge. This evidence should be widely communicated with the general public and global health community to prevent the psychological consequences of COVID-19 in different population groups. Nonetheless, it is essential to identify highly vulnerable individuals and connect them to required care, whereas access to mental health services and resources should be promoted, aiming positive mental health outcomes and psychosocial resilience across populations. This review provides an overview of early research on mental health epidemiology and encourages extensive research and evidence synthesis in the future, exploring the severity and causality of mental health problems. Lastly, evidence-based policymaking and practice should be adopted to guide how those mental health challenges can be mitigated in different contexts amid COVID-19 pandemic and future public health emergencies.

Pre-print: A previous version of this article is available as a preprint from PsyArXiv: https://doi.org/10.31234/osf.io/q8e5u ^76^

## Data availability

### Underlying data

No data are associated with this article.
