# Supplementary material for: Epidemiology of mental health problems in COVID-19: a review
Source: F1000Res. 2020 Jun 23;9:636. [Version 1] doi: 10.12688/f1000research.24457.1 (PMC7549174; doi:10.12688/f1000research.24457.1)
Supplement: Peer review history

# Peer review: Reviewer response for version 1

**DOI:** 10.5256/f1000research.26979.r69817

**Published:** 2020-10-09

**Authors:** Reindolf Anokye

**Affiliations:** 1 Edith Cowan University, Joondalup, Australia

Thank you for giving me the opportunity to review this article reporting the epidemiology of mental health problems in COVID-19. This is an important well-written article that will contribute to the literature in public health. The authors have done a great job putting this together and I would recommend that this review is published.

 I would also recommend that authors take their time to go through the article and correct any grammatical errors in the manuscript.

 Also, words such as psychological impact, psychological dimensions and psychological stress should not be used interchangeably with mental health if it is not probably a direct quote. For example, the sentence “psychological dimensions of COVID-19 are yet to be understood” could be rephrased as the mental health burden of COVID-19. Another statement “COVID-19 may have impacted psychological health among COVID-19 positive individuals” could also be rephrased. The consistency in the usage of mental health throughout the manuscript will make it easier for readers who are not expertise in this area to understand what the article is reporting.

 Furthermore, please indicate in the limitations section that most of the included studies were conducted in countries/places that reported most of the early cases of COVID-19.

Is the review written in accessible language?

Yes

Are all factual statements correct and adequately supported by citations?

Yes

Are the conclusions drawn appropriate in the context of the current research literature?

Yes

Is the topic of the review discussed comprehensively in the context of the current literature?

Yes

Reviewer Expertise:

health psychology,mental health,public health.

I confirm that I have read this submission and believe that I have an appropriate level of expertise to confirm that it is of an acceptable scientific standard.

# Peer review: Reviewer response for version 1

**DOI:** 10.5256/f1000research.26979.r71938

**Published:** 2020-10-07

**Authors:** Madison W Silverstein, Sarah Alonzi

**Affiliations:** 1 Loyola University , New Orleans, USA; 2 Department of Psychological Sciences, Loyola University New Orleans, New Orleans, USA

**Review of Epidemiology of mental health problems in COVID-19: a review.**

 Thank you for the opportunity to review this manuscript reporting the prevalence of depression, anxiety, and other adverse mental health effects of the COVID-19 pandemic. This is an interesting narrative review, with useful recommendations for researchers and clinicians. Minor revisions are needed to make findings and implications clearer to readers. I hope these suggestions can be of help! 

**Abstract**

 The abstract contained minor grammatical and sentence structure issues, which could be remedied as suggested.

- Two sentences in the abstract, (1) “the current literature” through “behavior” and (2) “moreover” through “survival”, are very long. These sentences can be broken up, or focus on a few of these factors (that authors deem most important for research and practice) to make the focus of the paper clearer to readers.
- Remove “on” from “future epidemiological studies should emphasize on psychopathological variations”

**Introduction**

- Paragraph 2, sentence 2. I found the sentence hard to read. It could be improved by changing persistence stress to persistent stress.
- Paragraph 2, final sentence. The fact that the review was “rapid” does not need to be mentioned and playing up the rapid peer review may undermine readers’ confidence in the validity of those findings.
- Paragraph 3, sentence 1. Change individuals who are tested positive to individuals who have tested positive.
- Paragraph 4, sentence 2. “As COVID-19 cases are impacting the capacities of health systems” is too vague. Include the way that they are impacting the capacities (e.g., adversely).

**Literature review on the epidemiology of mental health problems in COVID-19 **

- General population. In this section, it is unclear to me whether you are referring to the “general population” of a specific region, country, etc., or just summarizing existing population-level studies across locations and cultures. I would assume there is some variation in population-level stress across locations, so the term general population might be too broad. In addition to the heading, the body would benefit from more description of the countries and populations these reviews are focused on.
- Paragraph 5, final sentence. The sentence “furthermore, more than 80% of participants in this study perceived a need for mental health services,” is unclear. Did 80% perceive that they needed MH services, or that there should be MH services made available for the general population to cope with COVID-19?
- Patients with COVID-19. Removed were from “Furthermore, suicidal behavior among individuals who were tested positive”.
- Paragraph 9, sentence 5. The sentence, “moreover, altered consciousness in 21% of patients who subsequently died as reported in another study,” is confusing and needs revision.

**Factors associated with mental health problems during COVID-19 **

- Marital status. As you did in the previous paragraphs, it is necessary to state what marital status is associated with better or worse mental health outcomes.
- Comorbid mental health problems. The statement, “also, people with substance use disorders are susceptible to COVID-19, which may also increase their vulnerability to subsequent mental health problems” was quite confusing to me. The phrasing makes it seem as if people with substance use disorders are more susceptible than others, which id not supported by the literature.

**Discussion**

- Emphasizing on research and evidence mapping on mental health. The sentence starting with “perhaps, those countries” and ending with “on mental health during COVID-19” should be rephrased. Perhaps is unnecessary in this context, as data show that the countries listed in the previous section did, in fact, begin seeing COVID-19 cases later than China and Europe.

**Limitations **

- I would recommend adding that most of the literature cited in your review emerged from studies in China at the beginning of what became a global pandemic. As such, these findings might not be consistent across other affected countries and it is unclear how these mental health trends are developing as the pandemic continues.

Is the review written in accessible language?

Partly

Are all factual statements correct and adequately supported by citations?

Yes

Are the conclusions drawn appropriate in the context of the current research literature?

Yes

Is the topic of the review discussed comprehensively in the context of the current literature?

Yes

Reviewer Expertise:

Mental health during COVID-19, PTSD, health psych

We confirm that we have read this submission and believe that we have an appropriate level of expertise to confirm that it is of an acceptable scientific standard.
